# Oral Mucosa and Nails in Genodermatoses: A Diagnostic Challenge

**DOI:** 10.3390/jcm10225404

**Published:** 2021-11-19

**Authors:** Tiziana Cantile, Noemi Coppola, Vito Carlo Alberto Caponio, Daniela Russo, Paolo Bucci, Gianrico Spagnuolo, Michele Davide Mignogna, Stefania Leuci

**Affiliations:** 1 Oral Medicine Unit, Department of Neuroscience, Reproductive and Odontostomatological Sciences, University of Naples Federico II, 80131 Naples, Italy; tizianacantile@yahoo.it (T.C.); gspagnuo@unina.it (G.S.); mignogna@unina.it (M.D.M.); stefania.leuci@unina.it (S.L.); 2 Department of Medicine, Surgery and Dentistry Scuola Medica Salernitana, University of Salerno, 84081 Baronissi, Italy; 3 Department of Clinical and Experimental Medicine, University of Foggia, 71100 Foggia, Italy; vitocarlo.caponio@unifg.it; 4 Department of Advanced Biomedical Sciences, Pathology Section, University of Naples Federico II, 80131 Naples, Italy; danielarusso83@yahoo.it; 5 Department of Public Health, Section of Hygiene, University of Naples Federico II, 80131 Naples, Italy; paolo.bucci@unina.it

**Keywords:** genodermatoses, genetic diseases, oral mucosa, nails, dermatology, oral medicine

## Abstract

Genodermatoses represent a group of uncommon, hereditary, single-gene skin disorders, characterized by multisystem involvement, heterogeneous clinical manifestations and different degrees of morbidity and mortality. Some genodermatoses may have oral mucosa and nail involvement, since the oral cavity and cutaneous organ system, including nails, share a close embryologic origin. Nail disorders can manifest with nail hypoplasia or nail hypertrophy. Clinical pictures of affected oral mucosa can be extremely heterogeneous, ranging from asymptomatic papules to painful blisters, leukokeratosis, oral papillomas and fibromas to oral potentially malignant disorders and cancerous lesions. Oral mucosa and nails pathological features may occur synchronously or not and are usually associated with other systemic and skin manifestations. In some cases, oral mucosa and nails diseases may be distinct and constitute the principal sign of the genetic disorder, in other cases they represent only a part of the puzzle for the confirmation of the diagnosis. Continued awareness of the correlation between oral mucosa and nails findings can help physicians to diagnose genodermatosis in a timely manner, allowing more effective clinical management and prevention and/or early detection of complications. This article provides an overview of all specific genodermatoses affecting both oral mucosa and nails. Moreover, the correlation between teeth and nails is summarized in tabular form.

## 1. Introduction

Genodermatoses represent a group of uncommon, hereditary, single-gene skin disorders, characterized by multisystem involvement with heterogeneous clinical manifestations and different degrees of morbidity and mortality [1]. The molecular bases of genodermatoses include the following: mutations in structural epidermal genes; mutations in genes encoding integral components of the supramolecular adhesion complexes; and mutations in genes implicated in DNA repair pathways [2]. In the last years, advances in human genetics have allowed the discovery of new genes associated with inherited skin diseases, as well as revising definitions and categorizations for genodermatoses [3,4].

However, identifying hereditary skin disorders can be challenging because of both the rarity of these conditions and the lack of awareness, particularly when the skin disorder is not detectable at birth [5].

As timely diagnosis may provide an adequate clinical treatment of the disease itself and of the systemic manifestations, constant consciousness of the associations between a specific genetic mutation and the corresponding phenotype will enable more effective management of genodermatoses [4].

**Figure 1:**
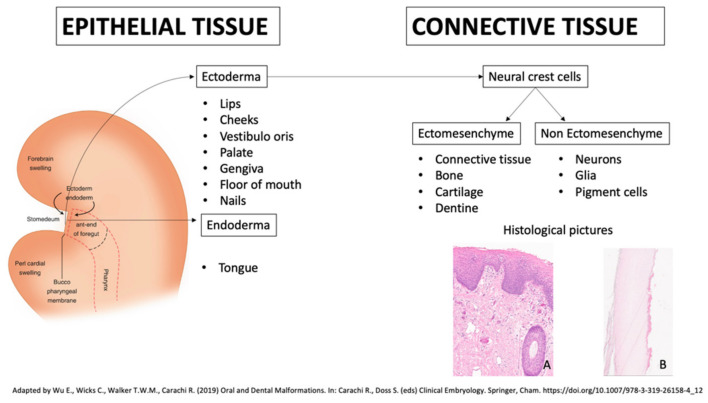
Embryogenesis and histological aspects of the oral mucosa and nails. (**A**) Normal squamous non keratinizing stratified epithelial lining of oral cavity mucosa connected to underlining chorion by the rete ridges. It consists of three layers: basal line, stratum spinosum and stratum corneum (haematoxylin and eosin, original magnification, 10×). (**B**) In a nail, the basal layer produces keratinocytes which differentiate (they flatten, lose the nucleus and become hard) to form the nail plate, shown here. An important histologic feature is the lack of a granular layer (haematoxylin and eosin, original magnification, 10×) (the slides were digitized with an Aperio AT2 scanner with 40× optics).

Within the large spectrum of clinical manifestations, since oral mucosa and cutaneous organ system, including nails, share a close embryologic origin (Figure 1) [6], it is possible to categorize a group of genodermatoses in which both oral and nails findings are present [1].

The oral mucosal epithelium develops for the most part from ectoderm (lips, cheeks, vestibule, palate, gingivae and floor of mouth) and also from endoderm (tongue). The oral mucosal connective tissue is originated from ectomesenchyme. At first, one layer of epithelial cells covers up the oral cavity; then, two cell layers develop at approximately five to six weeks. Afterward, the ectomesenchyme is initiated to secrete extracellular fibers. At 10 weeks, a multilayered epithelium can be observed. In the connective tissues, capillary buds and collagen start to show up. Epithelial proliferation and differentiation last over several weeks. At about 23 weeks in utero, an oral epithelium with adult features is present, consisting in a stratified ortho/parakeratinized palatal and gingival epithelium and a stratified non-keratinizing epithelium of the lips, cheeks, soft palate, ventral surface of tongue and floor of mouth [6]. Nail embryogenesis begins around week 10, and fingernail development precedes toenail development by around 4 weeks; fingernails reach fingertips after about 32 weeks and toenails after 36. The nails originate from a thickened ectodermal area located at the apex of the fingers. This thickening spreads over the dorsal surface of the fingers, forming the nail bed and surrounded by epidermal folds. The germinative layer of the proximal fold grows over the nail bed and keratinizes, forming the horny lamina. At the beginning of development, the nail is covered by a thin epidermal band called eponychium, which subsequently degenerates and leaves the nail uncovered except at the base where it persists in covering the nail, which forms the cuticle [7].

In genodermatoses, nail disorders can manifest either with nail hypoplasia or nail hypertrophy [7]. Furthermore, nail signs can be specific or nonspecific; involve one or more nails; occur simultaneously or secondary to systemic disease; present as alterations in shape, size, colour, or texture; and can be the primary site of affection or just a merely part of the disease [8,9].

In the oral cavity, a wide spectrum of diseases occurs due to genodermatoses, affecting both hard and soft tissues (oral mucosa, dentition and salivary glands) [10]. In particular, the clinical picture of affected oral mucosa can be extremely heterogeneous, ranging from asymptomatic papules to painful blisters, leukokeratosis, oral papillomas and fibromas to oral potentially malignant disorders and cancerous lesions [1].

Therefore, some genodermatoses may present with both oral mucosa and nails findings, occurring synchronously or not, which is usually associated with other systemic and skin manifestations [11]. In addition, sometimes oral mucosa and nails diseases represent the first clue of underlying genetic disorders; in other cases, they may be only a part of the puzzle for the confirmation of the diagnosis [9,10,12].

Actually, the definitive diagnosis of any genodermatosis is possible by using genetic tests, but be aware that the presence of specific oral mucosa and nail clinical findings may help clinicians who deal with oral cavity in differentiating genodermatoses from other diseases, in making a prompt diagnosis and, eventually, in referring the patient to adequate specialists [1,8].

The purpose of this article was to provide an overview of all specific genodermatoses affecting both oral mucosa and nails: Darier’s disease, dyschromatosis universalis hereditaria, dyskeratosis congenita, focal dermal hypoplasia, epidermolysis bullosa, pachyonychia congenita, Papillon–Lefèvre syndrome and tuberous sclerosis complex. Instead, genodermatoses affecting both teeth and nails are reported in Table 1.

Searches were conducted by using MEDLINE/PubMed and Web of Science. Only nails and oral mucosa diseases connected to genodermatoses were selected. Nails and oral alterations related to autoimmune, immune-mediated, chronic inflammatory, infective, drug-induced, deficiency states diseases, acute hypersensitivity reactions and acquired disorders were excluded from the present review.

For each disease, the pattern of inheritance, implicated gene, prevalence, diagnostic methods, onset, pertinent clinical oral mucosa and nails features, skin involvement, systemic manifestations, histopathological findings, differential diagnosis, overall treatment and prognosis are provided.

Oral findings, nails findings and differential diagnoses for each disease are summarized in Table 2.

### 1.1. Darier’s Disease

Darier’s disease (DD) (or keratosis follicularis) is an uncommon autosomal dominant genodermatosis with high penetrance and variable expressivity [31].

The prevalence of DD is 1:100,000 [32]. The onset is usually in the first or second decade of life [33].

The disease consists of keratotic papules occurring in the scalp, face, chest and back, nail dystrophy, palmar pits, verrucous papules on the dorsal hands and oral mucosal lesions [34].

Darier disease is generally diagnosed by the characteristics of the skin and family history, but genetic tests can be useful for confirming the diagnosis [35]. In fact, a mutation in the ATP2A2 gene, encoding sarco/endoplasmic reticulum calcium ATPase protein (SERCA2), causes Darier’s disease [32].

Affected individuals show red-brown hyperkeratotic papules, typically localized in the seborrheic zones (scalp, forehead, retro-auricular folds, upper arms, front and back of the central trunk and flexures). Confluent papules may result in verrucous or vegetating plaques. The lesions are often malodorous due to frequent bacteria, yeast and dermatophytic fungi secondary infections. Heat, sweating, sunlight and stress may exacerbate the disease [31,33]. Other cutaneous findings include palmoplantar pits, verrucous papules on the dorsal hands, acral keratoses and flat-topped papules [36].

**Figure 2:**
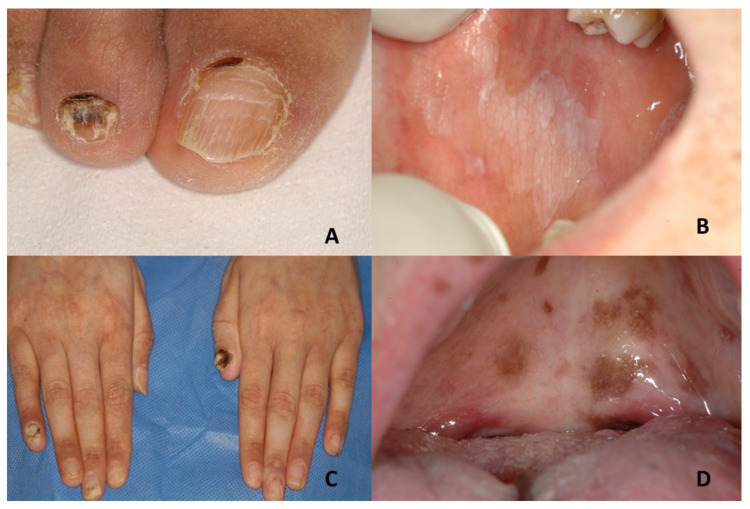
(**A**,**B**) Darier’s disease: dystrophic changes with streaks on toenails and papular keratotic lesion with a cobblestone appearance on the right side of buccal mucosa. (**C**,**D**) Dyschromatosis universalis hereditaria: dystrophic fingernails and hyperpigmented macules with mottled appearance on the hard and soft palate.

Nail findings include V-shaped notching at the distal end of the nail plate, subungual hyperkeratosis and red and white lines that are longitudinally oriented over the nail [32,36,37] (Figure 2A).

Oral lesions are characterized by asymptomatic several white papules in the buccal mucosa, hard palate and gingiva, resulting in a cobblestone appearance [31] (Figure 2B).

Neuropsychiatric features, including mental retardation, schizophrenia, bipolar disorder and epilepsy, can be present, as well [31].

On histopathology, dyskeratosis with corps ronds, acantholysis and hyperkeratosis can be observed [32].

This disease continues throughout life in a relapsing-remitting manner. Avoiding sunlight, heat, occlusive clothing and friction and using the appropriate treatments can permit partial control [32].

### 1.2. Dyschromatosis Universalis Hereditaria

Dyschromatosis universalis hereditaria (DUH) is an uncommon form of genodermatosis for which its exact prevalence is unknown [38] and is characterized by diffuse symmetrically distributed hypopigmented macules mixed with hyperpigmentation, with a reticulated pattern, involving almost all zones of the body, particularly the face [39]. Oral mucosa, hair and nails have also been involved [40]. The disease usually starts at infancy or early childhood, stops spreading before adolescence and persists throughout life without significant changes. Diagnosis is based on clinical features [41].

DUH is usually an autosomal dominant disease, with a small number of autosomal recessive and sporadic cases reported [42].

ABCB6, a member of ATP Binding Cassette (ABC) transporters, has been recognized as the first pathogenic gene connected with DUH [43].

Mutations of ABCB6 result in abnormalities in copper homeostasis, causing dysfunction of tyrosinase and further result in abnormal melanin synthesis. The ABCB6 gene is also involved in melanosome transport to surrounding keratinocytes [44].

However, DUH is a heterogeneous disorder and not all DUH patients have the ABCB6 mutation [43]. Recently, mutation in gene SASH1, which mediates skin melanogenesis, has been identified as a plausible cause of DUH [45].

When involved, oral mucosa and tongue can show mottled pigmentation [46]. Leukokeratosis of buccal mucosa, palatal mucosa and tongue has also been described [47]. Nails can appear dystrophic, with a few showing pterygium formation [47] (Figure 2C,D).

Histopathology of the skin reveals increased melanin in the basal layer and, rarely, melanin incontinence in the hyperpigmented lesions. The hypopigmented lesions display reduced melanin deposition in the basal layer. Histopathology from oral mucosa shows focal dyskeratotic changes [40,47].

Furthermore, DUH has been reported to be connected with a wide range of cutaneous and systemic diseases: tuberous sclerosis, learning difficulties, mental retardation, short stature, high tone deafness, X-linked oculo-cutaneous albinism, epilepsy, erythrocyte, platelet and tryptophan metabolism abnormalities, renal failure and primary ovarian failure [43].

To date, no useful therapy for hyperpigmented and hypopigmented lesions has been established [39].

### 1.3. Dyskeratosis Congenita

Dyskeratosis congenita (DC) is an uncommon inherited genodermatosis, first reported by Zinsser in 1906 [48]. Then, Engman and Cole described other cases; therefore, it is also known as the Cole–Engman syndrome or Zinsser–Cole–Engman syndrome [49].

DC is characterized by a classic triad: nail dystrophy, reticulate skin pigmentation over the trunk and neck and oral leucoplakia [50]. The assessed prevalence is under one per million and is extensively distributed among all races [51].

Diagnosis is based on clinical features; genetic testing may be useful for confirmation [52].

Dyskeratosis congenita is generally inherited in X-linked recessive form, which affects mainly males, but autosomal-dominant and autosomal-recessive cases have been described [50,53].

Pathogenesis is related to an inability to preserve the length of telomeres. In fact, mutations of genes encoding the RNA template (TERC), telomerase catalytic reverse transcriptase (TERT) and dyskerin, a component of the telomerase complex, have been observed in several DC families [54].

Therefore, tissues with a high cell turnover are most affected in DC [50].

Histologic features of DC are aspecific. Generally, there are telangiectasias observed superficially, with hyperkeratosis and atrophy of the epidermis. Many melanophages can be observed in the superficial dermis. Rarely, there is a vacuolar change in the basal layer of the epidermis, accompanied by a mild interface lymphocytic infiltrate. Fibrotic changes can be appreciated in the superficial dermis [55]. In addition, histopathologic examination of the intraoral lesions is frequently unhelpful, because DC can rarely be diagnosed by light microscopy [50].

The clinical findings of DC frequently appear in childhood and follow a classic model: nail dystrophy starts at 6 years, oral white plaques at 7 years and abnormal reticulate skin pigmentation at 8 years of age [50].

**Figure 3:**
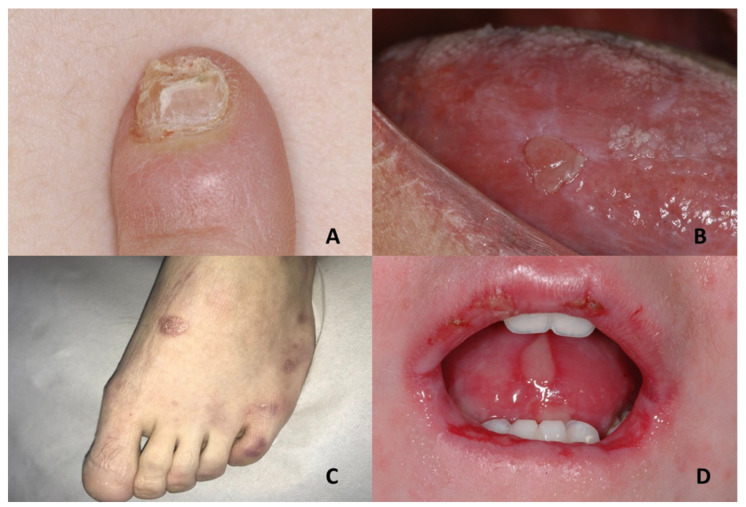
(**A**,**B**) Dyskeratosis congenita: dystrophic fingernail and keratotic, erythematous and ulcerative lesion of the right ventral tongue. (**C**,**D**) Epidermolysis bullosa: absent big toenail and multiple blistering lesions on the upper and lower labial mucosa and on the dorsal and ventral tongue. In the labial area, there are also micro-erosion and impetigo.

Nail dystrophy, appearing first on fingers and later on toes, starts with ridging and longitudinal splitting (Figure 3A). Gradual atrophy, thinning, pterygium and deformation result in small, undeveloped or missing nails [56].

Oral leukoplakia affects 80% of DC patients and may involve the buccal mucosa, tongue or palate, with the tongue being the most frequently involved [49]. At the beginning, lesions may appear as recurring vesicles and erosions, followed by white keratotic patches that result in erythroplakia with frequent episodes of ulceration (Figure 3B). Unhealed ulceration, infiltration and/or progressive expansion of oral mucosal lesions must be suggestive for the evolution into squamous cell carcinoma [51].

Lichen planus and lichenoid lesions are also described [49,56].

In addition to mucosal diseases, other oral signs include the following: periodontitis, alveolar bone loss, hypondontia, hypocalcification, thin enamel structure, taurodontism, short blunted roots and severe dental caries [51,53,56].

The skin of face, neck, chest, proximal limbs and intertriginous areas usually presents reticulated brown pigmentation, occasionally with superimposed discrete atrophy and telangiectasia, causing a poikilodermatous aspect [51]. Other described skin abnormalities include the following: alopecia of the scalp, eyebrows and eyelashes; early greying of the hair; hyperhidrosis; hyperkeratosis of the palms and soles; and loss of dermal ridges on fingers and toes [56].

Systemic manifestations appear with variable occurrences and include the following: growth and mental retardation, deafness, transparent tympanic membranes, lacrimal duct obstruction, conjunctivitis, blepharitis, microcephaly, amyloidosis, peripheral neuropathy, oesophageal stenosis, urethral stenosis, liver disease, pulmonary fibrosis, hypospastic testes, hypospadias, phimosis, horse-shoe kidneys, choanal atresia, frontal lobes atrophy, extensive intracranial calcifications, hypogonadism, osteoporosis, avascular necrosis of bone, immunological disturbances, bone marrow failure, aplastic anaemia and pancytopenia [51,56].

An increased presence of malignancies was also described in DC patients, including the following: squamous cell carcinoma of the mouth; nasopharynx; oesophagus; rectum; vagina or cervix; Hodgkin lymphoma; adenocarcinoma of the gastrointestinal tract; and bronchial, laryngeal and pancreatic carcinoma [51,56].

No effective and curative treatment is available for DC. The specific treatment for DC-related complications must be personalized to each patient [49,57].

Complications of bone marrow insufficiency, including aplastic anaemia, together with predisposition to malignancies are the main causes of death in DC patients, which happens at an average age of 24 to 30 years [58].

### 1.4. Epidermolysis Bullosa

Inherited epidermolysis bullosa (EB) represents a group of genetic diseases with skin fragility and blistering or erosions of the skin and mucous membranes as result of minimal trauma [59]. The prevalence is 1–9: 1,000,000 [60].

EB is generally classified into four types, which vary in terms of severity, in relation to the layer of skin in which blistering occurs: epidermolysis bullosa simplex (EBS) (with blisters within the epidermis); junctional epidermolysis bullosa (JEB) (blister formation occurs in the lamina lucida); dystrophic epidermolysis bullosa (DEB) (blister formation occurs below the lamina densa); Kindler syndrome (KS) (cleavage can occur at any level except in suprabasal layers) [61,62].

In addition, EB is stratified in more than 30 subtypes depending on clinical features, genetic inheritance, expression of the altered protein and genetic alteration [61].

To date, pathogenic variants in no less than 19 genes, encoding proteins essential for the integrity of cell–cell and cell–matrix association of keratinocytes such as desmosomes, have been identified, and more than 1000 mutations have been characterized as being associated with different EB subtypes [62].

The blisters on the skin can initially occur after birth or at any moment until early adulthood. They typically appear primarily at the zones of trauma or pressure (hands and feet) but may also be obesrved in mouth, esophagus, trachea, gastrointestinal and genitourinary tract and ocular mucous membranes. Furthermore, in some EB subtypes, changes in nails and hair can be observed [61,63]. (Figure 3C,D)

Diagnosis can be clinical on the basis of family history. Otherwise, a histological examination with immunofluorescence is necessary [63].

Epidermolysis bullosa simplex presents a large spectrum of clinical severity and a complex genetic background and is associated with mutations in seven distinct genes and includes at least 14 distinct clinical subtypes [61,64].

It is usually an autosomal dominant disease; autosomal recessive transmission in EBS is reported in only 5% of cases [62]. The common EBS subtypes comprise the following: localized, intermediate and severe forms [64]. In particular, in severe EBS characterized by large blisters grouped in an arch-shaped (herpetiform) distribution and usually associated with marked morbidity and mortality during the first years of life, the involvements of the oral mucosa and nail dystrophy are common [61,62].

Skin biopsy shows splitting within or above the basal cell layer [65]. In severe forms, the keratin intermediate filaments are clumped, which is a feature that constitutes a diagnostic finding [66].

*Junctional epidermolysis bullosa* is an autosomal recessive disorder characterized by mutations in seven different genes and stratified into nine different subtypes in relation to the clinical findings and the gravity of the phenotype [61]. The severity varies considerably across the two major subtypes, intermediate and severe, with the second associated with early mortality in the first two years of life [64]. In the severe subtype, a very significant finding is numerous symmetrically distributed granulation tissues in the mouth; central region of the face and nose; and the upper part of the back, axillas and inguinal folds. The clinical findings include oral blisters, microstomia and ankyloglossia. The intermediate subtype is characterized by blisters, atrophic scars and dystrophic or absent nails.

Skin biopsy shows splitting in the lamina lucida of the basement membrane of the epidermis or just above the basement membrane at the level of the hemidesmosomes in the lowest level of the keratinocytes layer. In the severe subtype, hemidesmosomes are hypoplastic and decreased in number; anchoring filaments are significantly reduced or missing. In the intermediate subtype, anchoring filaments may appear reduced; hemidesmosomes may appear reduced or hypoplastic [67].

*Dystrophic epidermolysis bullosa* comprises two large groups, dominant (DDEB) and recessive (RDEB), in relation to the genetic transmission; generally, RDEB is more severe than compared to DDEB [64]. Furthermore, DEB can be divided into no less than 11 clinical subtypes, according to the phenotypic characteristics involved [61].

In DEB patients the elementary oral mucosal lesion is a large size subepithelial blister that, once split, heals in fibrosis form. The fibrotic sequelae involve a reduced oral aperture (microstomia), ankyloglossia, loss of the vestibular fundus, lingual depapillation and atrophy of the palatal folds. Areas of leukoplakia have also been described, mostly on the lingual mucosa, as a result of the recurrent ulceration and re-epithelization phases [68]. Furthermore, most DEB patients have dystrophic fingernails and toenails, and the nails may be progressively lost [62].

Skin biopsy of all subtypes of DEB shows cleavage below the lamina densa of the basement membrane zone. In the recessive forms, anchoring fibrils are significantly reduced, missing or anomalous in shape. In dominant forms, anchoring fibrils may appear decreased in number and/or anomalous in shape; intracellular retention of collagen VII can be observed in some patients [69].

*Kindler syndrome* has a recessive autosomal inheritance. It is characterized by blistering at birth and followed by gradual development of poikiloderma, photosensitivity, premature skin aging and keratoderma. Atrophic scarring, nail dystrophy, contractures and interdigital synechia have been also observed.

Oral cavity can be involved, displaying the following factors: hemorrhagic mucositis, gingivitis, periodontitis, gingival hyperplasia, premature loss of teeth and labial leukokeratosis [70].

Complications of KS include severe colitis, esophagitis and urethral stenosis [62].

Histopathologic examination shows hyperkeratosis; aspecific epidermal atrophy; dermal edema; incontinence of pigment with or without cytoid bodies; loss of rete ridges; and focal vacuolization of the basal layer of the epidermis and pigmentary incontinence in the upper dermis, consistent with poikiloderma [71]. A distinctive finding is a split within the basement membrane zone at different levels [72].

Currently, there are no approved curative therapies for epidermolysis bullosa. Management is focused on prevention of blistering, wound care, pain alleviation, control of infection, nutritional support and prevention and treatment of extra-cutaneous complications. Prognosis depends on both EB subtype and general health of the patient [59].

### 1.5. Focal Dermal Hypoplasia

Focal Dermal Hypoplasia (FDH) or Goltz syndrome is a genetic skin disorder belonging to the ectodermal dysplasias [73].

It is a rare condition with about 300 cases reported, and its precise prevalence is unknown [74].

FDH is an X-linked dominant disease and is due to mutations in the PORCN gene (Xp11.23), which encodes the porcupine O-acyltransferase, implicated in the secretion and signaling of WNT proteins, which are essential for embryonic tissue development [74].

Diagnosis of FDH is usually made, according to clinical features in patients with classic ectodermal findings. Furthermore, molecular genetic tests may be helpful for validating the diagnosis [75].

Patients manifest cutaneous, nails, ocular, osseous, oral and dental defect [73].

FDH usually occurs in the neonatal period. In particular, skin findings can be observed at birth and are characterized as follows: atrophic and hypoplastic areas of skin; cutis aplasia; fat nodules in the dermis manifesting as soft, yellow-pink cutaneous nodules; and pigmentary changes. Histologic evaluation of mosaically affected FDH shows atrophic dermis, increased dermal adipocytes, increased papillary dermal capillaries, abnormal fibroblasts and sparse and disorganized collagen [76].

Verrucoid papillomas of the skin and mucous membranes, such as the mucosa of the mouth, nose, pharynx, larynx, trachea and esophagus, may appear later. Oral papillomas can be observed on the gingiva, tongue, palate, buccal mucosa and on histopathologic examination resembling squamous papillomas with hyperplastic, stratified squamous epithelium overlying a fibrovascular core in the absence of the typical morphologic evidence of human papilloma virus infection and stain negative for Epstein–Barr virus RNA [75]. Other oral soft tissue anomalies are represented by generalized gingivitis and intra-oral lipomas [74].

Dental defects can be present, such as enamel hypoplasia, hypodontia, oligodontia, taurodontism, abnormal tooth eruption, notching of the alveolar ridge, dental fusion and dental germination, supernumerary teeth, microdontia and abnormal root morphology [74].

**Figure 4:**
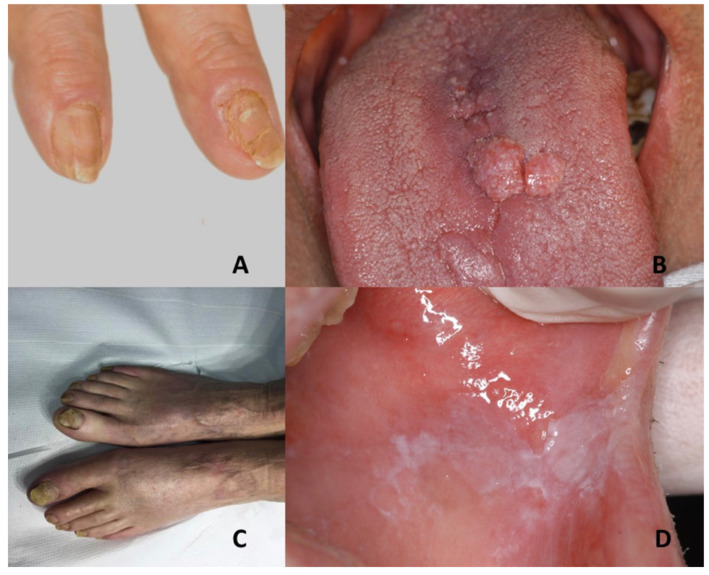
(**A**,**B**) Focal dermal hypoplasia: ridging and nails splitting and proliferative lesion with exophytic and sessile growth and verrucous surface on the dorsal tongue. (**C**,**D**) Pachyonychia congenita: hypertrophic onychodystrophy affecting all toe nails and oral leukokeratosis on the buccal mucosa on the left side.

Nails abnormalities can be present: longitudinal ridging of the nail plate of both the fingernails and toenails, associated with nail splitting. In addition, V-shaped notches at the distal free edge of the nail plate have been observed. Micronychia and anonychia can be also present [77] (Figure 4A,B).

Hair can be sparse or missing. Limb malformations comprise oligo-/syndactyly and split hand/foot. Anomalies of the eye can comprise the following: anophthalmia/microphthalmia, iris and chorioretinal coloboma and lacrimal duct abnormalities. Craniofacial features can comprise the following: facial asymmetry, notched alae nasi, cleft lip and palate and pointed chin. Other sporadic features may be present: abdominal wall defects, diaphragmatic hernia and renal anomalies. Psychomotor development is generally regular, but some patients show cognitive impairment [75].

There is no valid therapy for this disorder. Specific treatments must be applied according to patients’ clinical manifestations in order to improve specific function and cosmetic appearance [78]. FDH patients usually have a normal life expectancy; however, prognosis depends on the grade of severity [79].

### 1.6. Pachyonychia Congenita

Pachyonychia congenita (PC) is an uncommon genodermatosis with characteristic nail findings and other abnormalities of the palmoplantar skin, oral and laryngeal mucosae, teeth, pilosebaceous apparatus and hair [80]. The estimated prevalence is between 2000 and 10,000 cases of PC worldwide [81].

Pachyonychia congenita frequently occurs in infancy, but late-onset PC, known as pachyonychia tarda that appears in the fourth or fifth decade, has been described [82].

The pattern of inheritance is usually autosomal dominant with incomplete penetrance, but, infrequently, autosomal recessive and sporadic cases have been reported [83].

Today, PC has been categorized into five types according to the underlying keratins genes mutations (KRT6A, KRT6B, KRT6C, KRT16 and KRT17) resulting in structural alterations in keratin proteins [83,84,85].

Diagnosis is based on the clinical features and/or on the identification of a heterozygous pathogenic variant in one of the five keratin genes [86].

The most consistent clinical feature is hypertrophic onychodystrophy, one of the earliest signs of the disease, and it can appear within the first few months to years of life; however, sometimes, it presents later in life [86]. All nails are generally involved, although the findings are often most severe on thumbs, index fingers and toes, and the severity of the dystrophy differs from patient to patient [80,87].

No less than three phenotypes of hypertrophic nail dystrophy can be observed: nail grows to full length, but a distal prominent hyperkeratosis determines an upward slant with an accentuated curvature of the nail; the nail plate ends prematurely, leaving a distal region of hyperkeratosis and an exposed fingertip; the nail plate is thin with little or no hyperkeratosis [86] (Figure 4C). The histopathologic feature comprises marked hyperkeratosis of the nail bed [88].

Oral leukokeratosis often presents shortly after birth and can be considered one of the first signs of PC, mostly manifesting on tongue, buccal mucosa and, rarely, gingiva [87] (Figure 4D).

The tongue shows a white-to-yellow thickening that can be misdiagnosed as oral candidiasis, white sponge nevus and hairy tongue. Buccal leukokeratosis can mimic leukoplakia, but it is frequently accentuated along the bite line. Histologically, acanthosis and pronounced parakeratosis without a granular layer are observed [87]. Furthermore, angular cheilitis associated with secondary bacterial and yeast infection has also been described in PC patients [80]. Additional oral findings may include the following: enamel hypoplasia, neonatal teeth, hypodontia, periodontitis and severe caries [82].

With respect to cutaneous findings, palmoplantar keratoderma is the main one. It is a hard, non-erythematous, painful and focal keratoderma that is more pronounced in pressure points of the feet or in areas of chronic use on the hands, typically developing at the time a child begins ambulating and carrying weight [87].

On histopathology, gross hyperkeratosis with alternating orthokeratosis and parakeratosis can be observed. Acanthosis with patchy hypergranulosis in which large keratohyalin granules are present without gross epidermolysis is appreciable [89].

Other clinical PC features include the following: pilosebaceous cysts comprising widespread steatocystomas/steatocysts and vellus hair cysts, which frequently occur at puberty and continue during the course of adult life follicular keratoses in sites of friction; palmoplantar hyperhidrosis; excessive production of waxy material in the ear; intense and unexplainable ear pain; hoarseness due to laryngeal involvement; and coarse or twisted hair [80,86].

Similarly to most genodermatoses, there are currently no curative treatments. Therapy is directed at the manifestations that are most distressing to individual patients [80].

Prognosis depends on secondary infections, which are usually well controlled by antibiotics therapy [90].

### 1.7. Papillon–Lefèvre Syndrome

Papillon–Lefèvre syndrome (PLS) is a very uncommon autosomal recessive genodermatosis characterized by palmar plantar hyperkeratosis and severe periodontitis with early loss of deciduous and permanent dentition [91,92].

The prevalence is estimated between 1/250,000 and 1/1,000,000 [93].

PLS is caused by mutations in the CTSC gene encoding for cathepsin C (also called dipeptidyl peptidase I) and a lysosomal protease participating in epidermal differentiation and desquamation and in the activation of serine proteases expressed in cells of the immune system. CTSC mutations result in nearly complete inactivity of cathepsin C, which results in a deficiency in immunological response [91].

The clinical findings of the PLS appear between 1 and 5 years.

**Figure 5:**
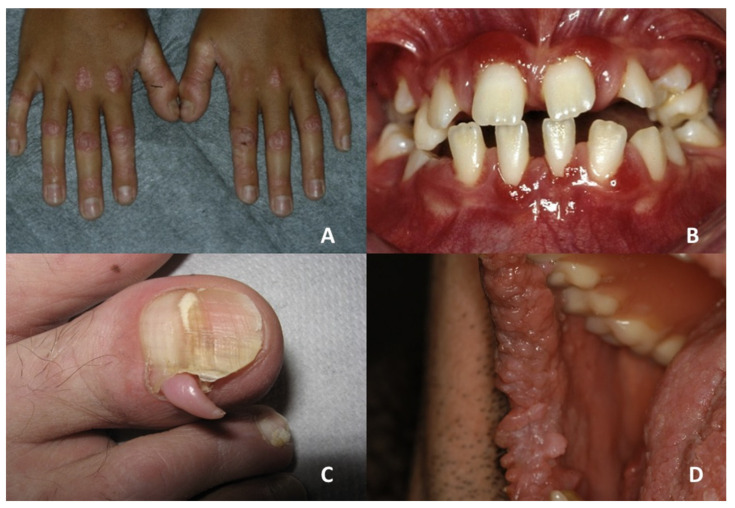
(**A**,**B**) Papillon–Lefèvre syndrome: clinical characteristics of affected skin on hands and severe periodontitis. (**C**,**D**) Tuberous sclerosis complex: ungual fibroma affecting a toenail and multiple fibromas in the right labial commissure.

Diffuse palmoplantar keratoderma with erythematous plaques can be observed, with the soles being frequently more involved than the palms (Figure 5A). Less frequently, the same skin lesions can be observed on the limbs (knees and elbows). Affected skin appears thick and red, with variable texture and colour; skin lesions exacerbate with low temperature, and walking can be painful [93].

On histopathology examination, skin of palms showed thickening of the epidermis, hypergranulosis, hyperkeratosis and mild mononuclear cell infiltrate of papillary dermis [91].

In the oral cavity, the major features are represented by severe gingivostomatitis and periodontitis (Figure 5B).

During the first year after eruption of deciduous teeth, gingiva becomes inflamed; after that, a rapid destruction of periodontium, in association with extensive bone resorption, deep periodontal pockets, lymphadenopathies, halitosis and early exfoliation of all deciduous teeth, is observed. The same sequence of events occurs when permanent dentition erupts [91,93].

On histopathology, gingival epithelium usually shows hyperkeratosis, acanthosis, hypergranulosis, occasional patches of parakeratosis, psoriasiform hyperplasia and inflammatory cell infiltrate (neutrophils, lymphocytes and plasma cells) [91,92].

Nail dystrophy can be present. Other features include the following: delayed somatic development, follicular keratosis, hyperhidrosis, calcification of falx cerebri and choroid plexus and predisposition to infections, such as furunculosis and respiratory tract infections [91].

Treatment is multidisciplinary, involving dermatologists, dentists and pediatricians. Dental management is a challenge: despite rigorous dental care, patients eventually become edentulous in the early adulthood. Lifespan is normal [92].

### 1.8. Tuberous Sclerosis Complex (TSC)

Tuberous sclerosis complex (TSC) is a multisystemic, neurocutaneous genetic disease characterized by hamartomas in multiple organs, including skin, kidney, lung and central nervous system [94].

The most frequent neurologic features are seizures; intellectual disability in different degrees; learning and behavioral problems; and autism [95].

This disease is usually identified in infants and children. However, due to the broad phenotypic variability, the disorder can be frequently unrecognized [96].

The prevalence is 1/20,000 [97].

Pattern of inheritance is autosomal dominant; nevertheless, two-thirds of TSC patients show the disease as a consequence of a de novo pathogenic variant [98].

Tuberous sclerosis complex is caused by mutations in genes TSC1 and TSC2 that encode hamartin and tuberin and is involved in the control of cell division and growth in the body [96]. In TSC patients, mutations in these genes cause a permanent activation of the mTOR pathway, which is responsible for cellular proliferation and inhibition of cellular apoptosis and, consequently, the formation of hamartomas in multiple organs [94].

Diagnosis is mainly based on clinical criteria, including the following major and minor features [96].

Major features include the following: hypomelanotic macules (more than two and at least 5 mm in diameter); angiofibromas (more than two) or fibrous cephalic plaque; ungual fibromas (more than one) (Figure 5C); shagreen patch; multiple retinal hamartomas; cortical dysplasias; subependymal nodules; subependymal giant cell astrocytoma; cardiac rhabdomyoma; lymphangioleiomyomatosis; and angiomyolipomas (more than one) [96].

Minor features include the following: confetti skin lesions; dental enamel pitts (more than three); intraoral fibromas (more than one); retinal achromic patch; multiple renal cysts; and non-renal hamartomas. Definitive diagnosis is established when patients show two major features or one major feature with at least two minor features, while “possible diagnosis” is recognized when patients show one major feature or at least two minor features [96].

Oral manifestations are frequently identified during childhood or puberty [99]. Oral fibromas are reported as multiple pink, small and fibrous nodules with a papillomatous appearance on the gingiva and less frequently on the buccal mucosa and dorsum of the tongue [1] (Figure 5D). Histopathology shows a dome-shaped papule with elongated rete; fibrosis; prominent fibroblasts; increased vascularity; and disseminated large, pleomorphic and stellate-shaped cells underlying thickened epithelium. Generally, calcifications or areas of ulceration are absent; significant collagenization can be observed [95]. Diffuse gingival overgrowth can also be present. Furthermore, multiple randomly distributed pits in dental enamel are reported [1].

Tuberous sclerosis can be correlated with cleft lip and palate, high-arched palate, bifid uvula and macroglossia. Cases of bony desmoid fibroids, odontogenic fibroids and myxomas have been described, with sporadic cases of oral angiomyolipoma [100].

To regard nails, ungual fibromas are detected frequently after the second decade of life in female patients and can gradually increase in volume, mostly affecting the toes [94].

Most ungual fibromas are pink to red, firm, conical papules developing from under the proximal nail fold. A longitudinal nail groove extends from under the tumour to the free edge of the nail. Histologically, they are non-encapsulated and comprise stellate shaped fibroblasts mixed with vertically oriented dense collagen and blood vessels. The lesions can be extremely vascular, predominantly fibrotic or show a mixed pattern. The epidermal modifications include compact hyperkeratosis, acanthosis, thickened granular layer and irregular rete ridges. Parakeratosis is not detected [101].

TSC is a life-long disorder. The treatment consists, mainly, in the management of the symptoms caused by hamartomas and prophylactic measures to avoid loss of functionality of the affected organs. Neurological and renal complications represent the main cause of morbidity and mortality. [94].

## 2. Conclusions

During the last decade, scientific knowledge on genodermatoses has been rapidly expanded. Nevertheless, making a correct diagnosis is still difficult, because of the rarity of these hereditary skin disorders, the high clinical heterogeneity, and, lastly, the little consciousness of these disorders among the medical community.

The present paper provides an extensive overview on genodermatoses in which oral mucosa and nails relevant findings can be useful as diagnostic key features.

Up to the present time and to the best of our knowledge, in the scientific literature this is the first paper aimed at describing and categorizing genodermatoses in which both oral mucosa and nails diseases are present.

Carrying out an inspection of the oral mucosa when a genetically determined nail disease is suspected and vice versa can help practitioners make diagnoses in a timely manner, allowing more effective clinical management and prevention and/or early detection of complications.

## Figures and Tables

**Table 1:** List of genodermatoses with both dental and nails findings.

Genodermatoses	Dental Features	Nails Features	References
Acro–dermato–ungual–lacrimal–tooth syndrome (ADULT syndrome)	Hypodontia	Dysplastic nails	Whittington et al., 2016 [13]
Rapp–Hodgkin syndrome (also known as: Ankyloblepharon-ectodermal defects-cleft lip/palate syndrome (AEC syndrome); Hay–Wells syndrome)	Hypodontia, abnormal tooth shape, dental caries and delayed dental eruption	Dystrophic nails	Tosun and Elbay, 2009 [14]
Schopf–Schulz–Passarge syndrome	Hypodontia	Dystrophic nails	Manchanda et al., 2017 [15] Khalil et al., 2020 [16]
Naegeli–Franceschetti–Jadassohn syndrome	Yellowish discoloration, abnormal dentition and enamel defects	Dystrophic nails	Shah et al., 2015 [17] Khalil et al., 2020 [16]
Ectrodactyly Ectodermal Dysplasia–Clefting syndrome (EEC syndrome)	Hypodontia and hypoplasia	Dystrophic nails	Bharati et al., 2020 [18]
Ellis–van Creveld syndrome	Conical teeth, enamel hypoplasia, hypodontia and natal teeth	Hypoplastic, dystrophic and friable nails	Lauritano et al., 2019 [19]
Witkop syndrome	Hypodontia	Spoon-shaped, rigid, slow-growing and friable nails	Devadas et al., 2005 [20]
Incontinentia Pigmenti	Dental shape anomalies, hypodontia and delayed dentition	Dystrophic nails	Khalil et al., 2020 [16] Nicolaou and Graham-Brown, 2003 [21]
Tricho-Dento-Osseous syndrome	Severe enamel hypomineralization and hypoplasia and taurodontic teeth	Thin, brittle nails	Khalil et al., 2020 [16] Islam et al., 2005 [22]
Hypohidrotic ectodermal dysplasia	Hypodontia or anodontia, small, conical and bulbous or taurodontic teeth	Thin nails	Reyes-Reali et al., 2018 [23]
Odonto-onychodermal dysplasia	Hypodontia, peg-shaped incisors and enamel hypoplasia	Dysplastic nails	Yu et al., 2019 [24] Khalil et al., 2020 [16]
Monilethrix (also known as: Moniliform hair syndrome)	Hypodontia and cone shaped teeth	Dystrophic nails	Khalil et al., 2020 [16]
Scalp–ear–nipple syndrome	Oligodontia and small peg-like teeth	Dystrophic nails	Naik et al., 2012 [25] Khalil et al., 2020 [16]
Oculodentodigital dysplasia	Hypodontia, defective enamel, abnormally small teeth and premature tooth loss	Brittle nails	Doshi et al., 2016 [26] Khalil et al., 2020 [16] Orphanet [27]
Rubinstein–Taybi syndrome	Talon-like cingulum on maxillary central incisors and hyperdontia	Racquet nails	Tirali et al., 2014 [28] Tosti et al., 2009 [29]
Porphyrias	Erythrodontia	Photo-onycholysis and marked koilonychias	Inamadar and Palit, 2012 [30]

**Table 2:** Oral findings, nails findings and differential diagnoses for each disease.

Genodermatosis	Oral Findings	Nails Findings	Differential Diagnosis
Darier’s disease	Asymptomatic multiple white papules with a cobblestone appearance	V-shaped notching at the distal end of the nail plate; subungual hyperkeratosis; red and white lines, longitudinally oriented over the nail	Acne vulgaris, seborrheic dermatitis, acanthosis nigricans, confluent reticulate papillomatosis, prurigo pigmentosa, reticulate erythematomucinous syndrome and acrokeratosis verruciformis of Hopf
Dyschromatosis universalis hereditaria	Mottled pigmentation	Dystrophic nails with a few showing pterygium formation	Xeroderma pigmentosum, dyskeratosis congenita, dyschromatosis symmetrica hereditaria, dyschromic amyloidosis, generalized Dowling-Degos disease, incontinentia pigmenti, Naegeli–Franceschetti–Jadassohn syndrome
Dyskeratosis congenita	Leukoplakia; evolution into squamous cell carcinoma	Nail dystrophy with ridging and longitudinal splitting. Gradual atrophy, thinning, pterygium and deformation, leading to small, undeveloped or missing nails	Nail-patella syndrome, twenty-nail dystrophy, keratoderma with nail dystrophy and motor-sensory neuropathy, poikiloderma with neutropenia, Fanconi anemia, Diamond-Blackfan anemia, Shwachman-Diamond syndrome, acquired aplastic anaemia, idiopathic pulmonary fibrosis
Epidermolysis bullosa	Epidermolysis bullosa simplex Large blisters grouped in an arch-shaped distribution Junctional epidermolysis bullosa Several symmetrically distributed granulation tissue in the mouth; blisters; microstomia; ankyloglossia Dystrophic epidermolysis bullosa Large size subepithelial blisters; microstomia; ankyloglossia; loss of the vestibular fundus; lingual depapillation; atrophy of the palatal folds Kindler syndrome Hemorrhagic mucositis; gingivitis; periodontitis; gingival hyperplasia; early dental loss; labial leukokeratosis	Epidermolysis bullosa simplex Nail dystrophy Junctional epidermolysis bullosa Dystrophic or absent nails Dystrophic epidermolysis bullosa Dystrophic or absent nails Kindler syndrome Dystrophic nails	Incontinentia pigmenti; ichthyosiform bullous erythroderma; epidemic pemphigus in new-borns; bullous ichthyosis of Siemens; toxic epidermal necrolysis; congenital porphyria
Focal Dermal Hypoplasia	Verrucoid papillomas	Longitudinal ridging of the nail plate associated with nail splitting; V-shaped notches at the distal free edge of the nail plate; micronychia; anonychia	Microphthalmia with linear skin defects, incontinentia pigmenti, TP63-related disorders, oculocerebrocutaneous syndrome, Rothmund-Thomson syndrome
Pachyonychia congenita	Oral leukokeratosis	Hypertrophic nail dystrophy	Onychomycosis, epidermolysis bullosa simplex, Clouston syndrome, amilial onychogryphosis, twenty-nail dystrophy, dyskeratosis congenita, palmoplantar keratoderma striata, Olmsted syndrome
Papillon–Lefèvre syndrome	Severe gingivostomatitis and periodontitis	Nail dystrophy	Haim-Munk syndrome, prepubertal/aggressive periodontitis, keratosis punctate, Greither’s syndrome, keratoderma hereditarium mutilans, leukemia, Takahara’s syndrome, localized epidermolytic palmoplantar keratoderma, Meleda disease, Howel–Evans syndrome
Tuberous sclerosis complex	Oral fibromas	Ungual fibromas	Vitiligo, nevus depigmentus, nevus anemicus, piebaldism, Vogt–Koyanagi–Harada syndrome, hypomelanosis of Ito, cardiac myxoma, isolated brain tumors, pulmonary emphysema, acne vulgaris, acne rosacea, or multiple trichoepithelioma

## Data Availability

Data is contained within the article.
